# The Miami Medical College

**Published:** 1865-07

**Authors:** 


### THE MIAMI MEDICAL COLLEGE

Has recently been reorganized, and preperations are being made by which the most thorough course of medical instruction will be given. A large and very strong corps of professors has been elected to fill the various chairs. With one thing we are highly gratified viz: that several of the chairs have been established and filled with reference to special departments of medical practice; this we think a move in the right direction. We think that it is a full well established, and generally admitted truth, that even a thorough knowledge of the general principles of medicine does not enable any one to practice many of its specialties properly and efficiently. The course of instruction in this institution will be given in the Ohio Dental College Building, where ample facilities are afforded for every particular. Many of the Professors are old and popular teachers; and all have been selected with particular reference to their ability in this respect. The design of those interested is to make the institution such an one as the profession in this age demands; and we are well assured that there is the ability in the faculty to make it such.

